# Fighting parasites and predators: How to deal with multiple threats?

**DOI:** 10.1186/1472-6785-12-12

**Published:** 2012-07-24

**Authors:** Olivia Hesse, Wolfgang Engelbrecht, Christian Laforsch, Justyna Wolinska

**Affiliations:** 1 Department of Biology II, Ludwig-Maximilians-University Munich, Grosshaderner Str 2, 82152, Planegg-Martinsried, Germany; 2 GeoBio Center, Ludwig-Maximilians-University Munich, Munich, Germany

**Keywords:** *Daphnia*, Host-parasite, Inducible defences, *Metschnikowia* sp., Multiple stressors, Phenotypic plasticity, Predator–prey

## Abstract

**Background:**

Although inducible defences have been studied extensively, only little is known about how the presence of parasites might interfere with these anti-predator adaptations. Both parasites and predators are important factors shaping community structure and species composition of ecosystems. Here, we simultaneously exposed *Daphnia magna* to predator cues (released by the tadpole shrimp, *Triops*, or by a fish) and spores of the yeast parasite *Metschnikowia* sp. to determine how life history and morphological inducible defences against these two contrasting types of predators are affected by infection.

**Results:**

The parasite suppressed some *Triops*-induced defences: *Daphnia* lost the ability to produce a greater number of larger offspring, a life-history adaptation to *Triops* predation. In contrast, the parasite did not suppress inducible defences against fish: induction (resulting in smaller body length of the mothers as well as of their offspring) and infection acted additively on the measured traits. Thus, fish-induced defences may be less costly than inducible defences against small invertebrate predators like *Triops*; the latter defences could no longer be expressed when the host had already invested in fighting off the parasite.

**Conclusions:**

In summary, our study suggests that as specific inducible defences differ in their costs, some might be suppressed if a target prey is additionally infected. Therefore, adding parasite pressure to predator–prey systems can help to elucidate the costs of inducible defences.

## Background

Inducible defences, which are found among various groups of organisms, can evolve when there is spatial or temporal heterogeneity in predation risk [e.g. [1,2]]. Often, such defences are triggered by predator-released chemical cues, so called kairomones [3] and may be additionally altered in response to hetero- and conspecific alarm cues [4,5]. Although beneficial when predation is high, inducible defences are assumed to come at a cost which could potentially be saved in predator free environments. If no costs exist, defences should be expressed permanently [e.g. [6,7]]. The costs to sustain such an adaptive defence system have been classified as different types: First, there might be costs related to the maintenance of sensory or regulatory systems needed to detect environmental conditions. Secondly, energy and resource investment might be needed for constructing, maintaining and operating the defensive traits. Further costs can arise from self damage (e.g. autotoxicity), opportunity costs like the long-term consequences of allocation or developmental constraints and finally, environmental costs (expressing a suboptimal phenotype in a given environment) [1,8]. Still, the existence, modality and extent of these costs are debated and many empirical studies have found only negligible to weak costs [e.g. [9,10]]; reviewed in 8. However, while costs may not be apparent under optimal conditions, there may be a reduction in fitness under conditions of stress [11].

Parasites are a common threat across ecosystems [12]. Thus, many organisms are simultaneously prey for predators and hosts for parasites [13]. Often the response to one stressor has further implications regarding an individual’s defence towards another stressor. For example, parasite-mediated alterations of anti-predator behavior have been shown in amphibians infected by fungi [14] and crickets parasitized with nematodes [15]. Freshwater snails respond to the presence of predatory crayfish with predator avoidance behavior but, at the same time, suffer a reduced ability to defend against potential pathogens [16]. Similarly, the long-term exposure of hamsters to an overdose of weasel odor can suppress the immune system [17] and likewise the immune defense of damselflies is suppressed under presence of fish predators [18]. Interestingly, another study in damselflies showed an increased investment into some components of the immune system in the presence of predatory dragonflies [19] suggesting that induction of the immune defence is differently affected depending on the type of predator the host is exposed to.

A suitable model organism to investigate the effects of parasites on inducible defences is the “water flea” *Daphnia*, a small planktonic crustacean. *Daphnia* is known to change its morphology, life history and behavior in response to predators (reviewed in [20]) and is also commonly studied in host – parasite interactions [e.g. [21,22]]. Although *Daphnia* are often exposed to predators and parasites simultaneously, there are few studies that have investigated the effects of infection on inducible defences. It has been shown that *Daphnia galeata* can still express inducible defences against fish, regardless of infection with protozoan or yeast parasites [23,24]. The same was true for *D. magna* infected with a bacterial parasite [25]. In contrast, simultaneous exposure of *D. magna* to the same bacterial parasite but to an invertebrate predator (phantom midge larvae) *,* resulted in antagonistic or additive effects on some host life history traits [26]. The different outcome of the two latter studies may have resulted from the different predator types involved.

In general, large predators prefer to hunt larger prey and thus exert a selective pressure for the prey to mature earlier at a smaller size, whereas small predators are limited to small prey and select for delayed prey maturity at larger size [e.g. [27,28]]. The dominant large predators on *Daphnia* are planktivorous fish. In response to fish kairomone, *D. magna* mature earlier at a smaller body size, develop elongated tail spines and produce more but smaller offspring e.g. [29,30]. Among the invertebrate predators of *D. magna* are the tadpole shrimps, *Triops*[31,32], which are limited to smaller prey by the size of their food groove and the opening width of the mandibles. *D. magna* respond to *Triops* by getting ’bulky’ (i.e. they increase in body length and width) and by developing elongated tail spines [31,33]. Since the *Triops*-induced defence contrasts the induced response of *Daphnia* towards fish, the influence of parasites on these two types of inducible defences might not be the same. Given the variability in *Daphnia’s* response to parasites and predators, it is difficult to elucidate general costs imposed by simultaneous exposure to both threats.

Our study aims to analyze the influence of parasites on the expression of the two contrasting anti-predator defence strategies and to compare potential costs of these defences. To investigate this question we infected *D. magna* with the parasite *Metschnikowia* sp. (family Hemiascomycetes, [34]), which causes major reductions in host life span and fecundity [35-37], and exposed them to two contrasting types of predators, fish and *Triops*.

## Methods

### Origin and care of host, parasite and predators

We tested a single *Daphnia magna* clone isolated from a temporary pond in Oxford, England. *Daphnia* were kept in climate chambers at 20 ± 0.5 °C with a constant photoperiod (15 h light and 9 h dark) in artificial medium (ultrapure water, phosphate buffer and trace elements) and were fed three times per week with green unicellular algae ( *Scenedesmus obliquus*). For three generations prior to the experiment, *Daphnia* were kept individually in 100 ml of medium which was exchanged every third day and fed daily with 2 mg Cl^-1^ of *S. obliquus*. The *Metschnikowia* sp. strain was isolated from lake Ammersee in Germany, and cultured on the same *D. magna* clone as used in the experiment. Two predators were tested: the tadpole shrimp, *Triops cancriformis*, and the fish, *Rhodeus amarus* (hereafter referred to as ‘ *Triops*’ and ‘fish’, respectively). A clonal line of *Triops* was provided by Dr. E. Eder (Zoological Institute, University of Vienna). Different size classes were raised separately in 8 l aquaria filled with semiarticifial medium (wellwater and aqua bidest. 1:1) and fed with *Chironomidae* and commercial fish food (Grana Discus, JBL GmbH & Co. KG, Germany) ad libitum. The fish were obtained from a commercial store; 20 individuals were kept in a 100 l aquarium and fed with commercial fish food. The experimental research on animals followed internationally recognized guidelines.

### Preparation of kairomone media

Three types of media were prepared daily: 1) *Triops* kairomone (a 2 l beaker was stocked with two *Triops* for 24 h; *Triops* size: 2–3 cm), 2) fish kairomone (a 5 l beaker was stocked with one fish for 24 h; fish size: 6–7 cm), and 3) control medium (no kairomone). The fish density was similar to that used in previous studies [e.g. [23,24]]. The applied *Triops* density is lower to that found in natural concentrations of this predator (up to 2500 Triops/m² in natural ponds, [38]) and adequate for defence induction in *D. magna *[31]. The predators were fed with a commercial fish food (preliminary experiments showed no effect of the fish food on the defence expression in *Daphnia*, CL, unpublished data); the same amount of fish food was added daily to the kairomone-free treatments. Additionally, predators were fed adult *D. magna* of the same clone as the experimental units: each *Triops* obtained approximately 15–20 *Daphnia* and each fish 30–40 *Daphnia* per day. Hence, the term ‘kairomone’ refers not only to cues released by the predators but also to alarm substances released from prey during their consumption by the predator [4]. Prior to use all media were filtered (0.22 μm).

### Experimental set-up

*D. magna* were individually exposed to predator kairomones and/or parasite spores. This resulted in six treatments, with 20 replicates each: one treatment without kairomones and without infection (‘control − no parasite’), one without kairomones but with infection (‘control − parasite’), two kairomone treatments without infection (‘ *Triops* − no parasite’ and ‘fish − no parasite’) and two double-stressed treatments (‘ *Triops* − parasite’ and ‘fish − parasite’). On day 1, third clutch newborns (< 24 h) from age-synchronized mothers were placed individually in 5 ml of *Triops*-, fish- or control-medium on a random basis. On days 1 and 3 a parasite spore solution (obtained by homogenization of the infected *D. magna*) was added at a concentration of 2200 (day 1) and 2800 (day 3) spores ml^-1^. A placebo solution was analogously prepared from the same number of uninfected *Daphnia* and given to the non-infected treatments. On day 2, 5 ml of medium was added and on day 3 the *Daphnia* were transferred into 10 ml of new medium. On day 4, an additional 10 ml of medium was added to all jars. From day 5 onwards, all individuals were kept in 40 ml of medium, which was exchanged daily (before, the medium was kept at a small volume to increase the probability of spore ingestion by *Daphnia*). The *Daphnia* were fed daily with 2 mg Cl^-1^ *S. obliquus* (except days 2 and 4, when only 1 mg Cl^-1^ was added). The experiment lasted 24 days, at which point all infected animals had died.

### Recorded parameters

We collected the following life history and morphological data: 1) age at maturity (i.e. the day of 1^st^ clutch release), 2) life span, 3) number of offspring in the first three clutches, 4) body length (distance between the upper edge of the compound eye and the base of the tail spine) after the release of each of three clutches, and 5) body length of five randomly selected offspring per mother from each of the first three clutches (average per clutch was used for statistical analyses). For the morphological measurements we used a digital image-analysis system (Cell^P, Olympus, Hamburg, Germany). Finally, when the *Daphnia* died the body length was measured and the concentration of mature parasite spores [39] was counted using a Neubauer Improved counting chamber.

### Statistical analyses

All analyses were performed with PASW Statistics (version 18.0). We used a two-way ANOVA with three levels of kairomone treatment (fish, *Triops* and control) and two levels of infection (parasite and no-parasite). Age at maturity and life span were transformed prior to analysis (Rankit transformation, [40]). A Tukey’s PostHoc Test was run to distinguish between the effects of the different kairomone treatments. Parasite spore load was analysed by ANCOVA (with *Daphnia* body length at death as a covariate) and the interaction of kairomone × body length was included in the model. Individuals from the parasite treatments which did not become infected (n = 4) were excluded from all analyses. Similarly, individuals which died before day 10 (i.e. day when infection was first detectable) were also excluded (n = 5).

## Results

### Age at maturity and life span

**Figure 1:**
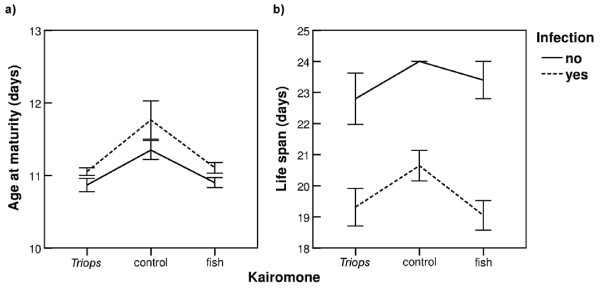
**Means (± SE) of (a) age at maturity and (b) life span across three types of medium (*Triops*kairomone, control, fish kairomone), and for both infected (filled symbols) and non-infected (open symbols)*Daphnia magna.***

Infected *Daphnia* matured significantly later than non-infected *Daphnia* whereas *Triops* and fish exposure led to earlier maturation regardless of infection status (Figure 1, Table 1). In addition, infection led to significant reductions in life span (Figure 1, Table 1).

**Table 1:** **The effects of infection and kairomone treatments on*Daphnia magna*life history and morphological traits (two-way ANOVAs)**

**Dependent variable**	**Clutch**	**Infection (df = 1)**		**Kairomone (df = 2)**		**Infection × Kairomone (df = 2)**	
		**F**	**p**	**F**	**p**	**F**	**p**
Age at maturity		7.7	**0.006**	14.3	**< 0.001 (F, T)**	0.01	0.993
Life span		124.1	**< 0.001**	3	0.053	0.4	0.657
Number of offspring	1	17.6	**< 0.001**	0.9	0.425	0.3	0.766
	2	38.3	**< 0.001**	1.9	0.156	3.5	**0.034**
	3	101	**< 0.001**	3.7	**0.029**	0.04	0.961
Body length	1	63.6	**< 0.001**	19	**< 0.001 (F, T)**	4.2	**0.017**
	2	124.8	**< 0.001**	18.7	**< 0.001 (F, T)**	1.4	0.261
	3	130	**< 0.001**	17.7	**< 0.001 (F, T)**	0.3	0.706
Offspring	1	14.7	**< 0.001**	12.3	**< 0.001 (F, T)**	0.9	0.411
body length	2	1,7	0.197	11.8	**< 0.001 (F)**	1.2	0.307
	3	70.9	**< 0.001**	18.8	**< 0.001 (F)**	3.7	**0.030**

Significant values are given in bold. The exact occurrence of significant differences between the kairomone treatments and the control are given as “F” or “T” for the fish and *Triops* treatment, respectively.

### Number of offspring

**Figure 2:**
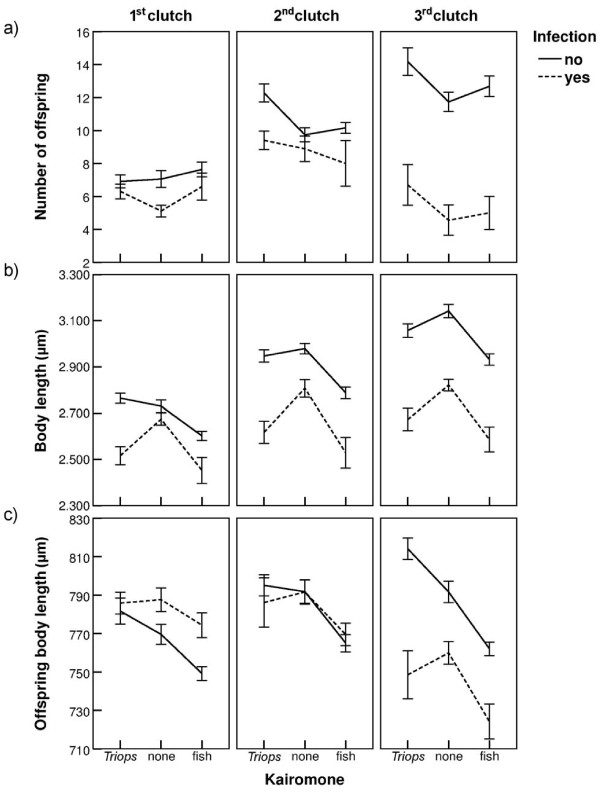
**Means (± SE) of (a) number of offspring, (b) body length and (c) offspring body length for 1**^**st**^**, 2**^**nd**^**and 3**^**rd**^**clutch, across three types of medium (*Triops*kairomone, control, fish kairomone), and for both infected (filled symbols) and non-infected (open symbols)*Daphnia magna*.The scale of the y-axis is the same across the three presented clutches.**

Infected *Daphnia* produced significantly less offspring than uninfected *Daphnia* (in the 3^rd^ clutch: ~ three times less; Figure 2a, Table 1). Moreover, none of the infected individuals produced more than three clutches, whereas the uninfected *Daphnia* produced five clutches by the end of the experiment. Regarding the kairomones, exposure to fish had no effect on the number of offspring, but *Triops*-exposed *Daphnia* produced more offspring than controls in the 2^nd^ and 3^rd^ clutch (Figure 2a). However, this effect was suppressed by infection (see infection × kairomone interaction in the 2^nd^ clutch, Table 1).

### Body length

Infection as well as fish kairomone exposure led to a significant decrease in body length across all three clutches (Figure 2b, Table 1). *Daphnia* exposed to *Triops* kairomones were smaller in size, but only when additionally infected (see significant interaction in the 1^st^ clutch, Table 1). The offspring of infected mothers were significantly larger in the 1^st^ clutch, but smaller in the 3^rd^ clutch (Figure 2c, Table 1). Although *Triops*-exposed mothers had larger offspring in their 1^st^ and 3^rd^ clutches, infection suppressed this effect in the 3^rd^ clutch (see significant interaction, Table 1). In contrast, fish-exposed *Daphnia* produced significantly smaller offspring in all clutches, independent of infection (Figure 2c, Table 1).

### Spore load

**Figure 3:**
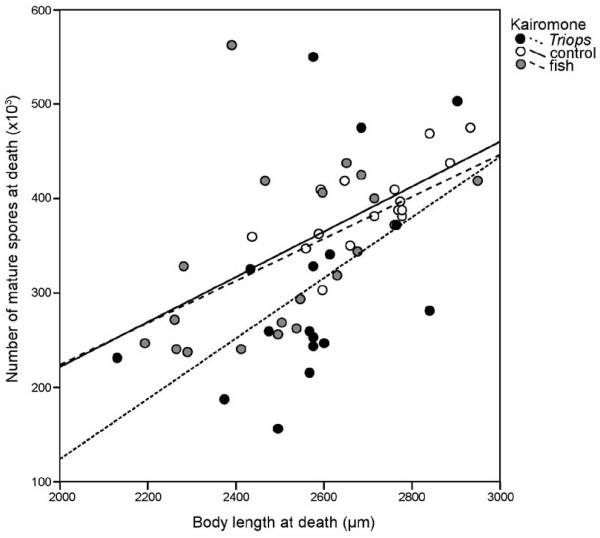
**Number of parasite spores counted for each infected*Daphnia magna*in relation to its body length at death.Trend lines are drawn for each type of medium (*Triops* kairomone, control, fish kairomone).**

Larger *Daphnia* contained significantly more parasite spores (F_1,53_ = 14.2; p < 0.001). However, kairomones had no effect on the amount of spores (F_2,53_ = 0.3; p = 0.708) and there was no significant kairomone × body length interaction (F_2,53_ = 0.2; p = 0.783; Figure 3).

## Discussion

Both tested predator cues had significant effects on the life history and morphology of *Daphnia magna*. When exposed to fish kairomones, *Daphnia* matured earlier, at a smaller body size and produced smaller offspring (as in [29,30]). *Triops*-induced *D. magna* also matured earlier (as in [31]) but at a similar size than the control *Daphnia*. In addition, their offspring were larger than control offspring. Previous studies on *Triops*-exposed *D. magna* reported an increase in body width and body length for induced individuals (i.e. “bulkiness”) [31]. The lack of such an effect in adult *Daphnia* in the present study might be caused by clonal differences in response to kairomones, such as have been reported from this [33] and other predator–prey systems [e.g. [41,42]]. In addition, the *Daphnia* were exposed to the kairomone only after they were released from their mothers’ brood pouch. This may account for the less pronounced inducible defences. Previous studies using other *Daphnia* species have shown that the sensitive phase for induction starts already during embryonic stages, resulting in the offspring from predator-exposed individuals being better defended (i.e. “maternally induced defence”) than offspring from unthreatened parents [43,44]. This seems to be concordant with the observation that the *Triops*-induced adult *Daphnia* did not show an increase in body length while their offspring were significantly larger compared to control individuals.

Regarding the effect of infection, similar to findings from other studies, *Metschnikowia*-infected *Daphnia* showed delayed maturity, produced fewer offspring, were smaller in size and died earlier [e.g. [35-37]]. It seems that the parasite consumes resources that could otherwise be invested into host reproduction and growth [36]. Surprisingly, the first-brood offspring of infected mothers were significantly larger than offspring of non-infected individuals. As the parasite shortens the host’s life span and its ability to reproduce, a larger investment into first-brood offspring may be a strategy to maximize the fitness of infected hosts; at least in the presence of invertebrate predators or at low predation risk. This corresponds to the observation that larger offspring are produced in response to unfavourable environmental conditions in *Daphnia* [e.g. [45,46]], as well as in other organisms [e.g. [47-49]].

It has been reported that exposure to fish kairomones can cause higher susceptibility to infection in another *Daphnia* species [24], and that *D. magna* have an increased risk of infection when they sink to lower depth to escape fish predation [50]. Conversely, other studies found that *Daphnia* resistance and/or parasite virulence remain unaffected by simultaneous exposure of the *Daphnia* host to fish kairmones [23,25]. In our study parasite spore load did not differ between predator-exposed and predator-naive *Daphnia.* Instead, we show that simultaneous exposure of *Daphnia* to parasites and predator kairomones can result in synergistic effects; this was most pronounced by the reduction in body length: double-stressed individuals were smallest (in all three clutches) and produced the smallest offspring (in the third clutch). The most interesting pattern in our experiment was the offspring body length, where the *Triops*-induced response (but not the fish-response) was suppressed by infection. A reduced body length may impose particularly high costs for *Triops*-exposed *Daphnia* as they need to grow large to be successfully protected against this invertebrate predator [31,33]. Moreover, there might be some other costly defences against *Triops* that have not been assessed in our study. It has been shown that *Daphnia* strengthen their carapace by developing a thicker armour as protection against this invertebrate predator [51], a response also observed for *Triops*-exposed *D. magna* (Rabus et al., in preparation). These aspects might explain why only the defences against *Triops*, but not against fish, were suppressed by additional parasite stress. The *Triops*-induced response seems to require more resources which might have already been invested into parasite defence. Indeed, raising the immune defence is considered costly for invertebrates [52]. In contrast, a reduction in body length results in an even stronger defence against fish predation [23-25] and for fish-induced *Daphnia*, remaining small and producing smaller offspring does not require additional resources. However, smaller *Daphnia* have a lower feeding rate and thus take up fewer resources than bigger individuals [53]. Moreover, smaller *Daphnia* are also morphologically limited by the size of their brood pouch and therefore produce smaller eggs [54]. Hence, the latter aspects may therefore account for a lower fitness also in the case of fish- and parasite-exposed *Daphnia*.

There might be costs involved in other traits that were not tested in this study. For instance, diel vertical migration, a behavioral defence response of *Daphnia* under fish predation [e.g. [55,56]], has been shown to be altered by parasite infection [57]. Moreover, since immune systems are highly plastic we cannot rule out that the investment into fighting off the parasite differed between the two predators the *Daphnia* were exposed to. In damselflies, for instance, it has been shown that risk of water mite parasitism and predation by dragonflies can increase investment into immunity [19]. However, since our study did not aim to measure the immune response it remains speculative if fighting off the parasite under different predator regimes results in a variable amount of resources available for the expression of defensive traits.

## Conclusions

In nature, the result of combined predator and parasite stress seems to be variable as has been shown solely for multipredator scenarios e.g. [58,59]. Here the development of each trait is assumed to depend on its benefits and costs in the current environment since investment into a specific defence in the context of varying stressors is always a trade-off [60]. The presence and extent of the costs of inducible defences are still being debated. Theoretical models assume that inducible defences should be costly, as organisms would otherwise be constitutively defended [e.g. [6,7]]. However, many empirical studies find only negligible or weak costs (reviewed in [8]). We think that adding parasite pressure to studies of predator–prey systems can be a useful tool to elucidate the nature and extent of these costs. Our results suggest that *Daphnia* which express inducible defences against smaller invertebrate predators suffer more from an additional stressor, here parasites, than *Daphnia* expressing inducible defences against large vertebrate predators, at least for the traits measured in this study. Further research on the interactions between parasites and inducible defences, including other levels of defence and also traits of the immune system is required in order to reveal general patterns. The simultaneous impact of different threats may have important effects on species interactions in natural ecosystems.

## Competing interests

There are neither financial nor non-financial competing interests involved in this study.

## Authors’ contributions

All authors were involved in designing the study. OH and WE performed the experiment and analysed the data. OH wrote the manuscript with the support of CL and JW. All authors read and approved the final manuscript.

## Authors information

Christian Laforsch and Justyna Wolinska share senior authorship.
